# Observation of a temperature dependent anomaly in the UV translucency of milk useful for UV-C preservation techniques

**DOI:** 10.1038/s41598-023-49124-y

**Published:** 2023-12-11

**Authors:** Jaayke L. Fiege, Benedikt Woll, Stefan Hebig, Alexandra Dabrowski, Volker Gräf, Elke Walz, Stefan Nöbel, Katrin Schrader, Mario Stahl

**Affiliations:** 1 Department of Food Technology and Bioprocess Engineering, Max Rubner-Institut, Federal Research Institute of Nutrition and Food, 76131 Karlsruhe, Germany; 2 Department of Safety and Quality of Milk and Fish Products, Max Rubner-Institut, Federal Research Institute of Nutrition and Food, 24103 Kiel, Germany

**Keywords:** Biophysics, Microbiology, Engineering, Nanoscience and technology, Optics and photonics

## Abstract

Milk fat globules and casein micelles are the dispersed particles of milk that are responsible for its typical white turbid appearance and usually make it difficult to treat with modern ultraviolet light (UV) preservation techniques. The translucency of milk depends largely on the refractive indices of the dispersed particles, which are directly affected by temperature changes, as incorporated triglycerides can crystallize, melt or transition into other polymorphs. These structural changes have a significant effect on the scattering properties and thus on the UV light propagation in milk, especially by milk fat globules. In this study, a temporary minimum in the optical density of milk was observed within UV wavelength at 14 °C when heating the milk from 6 to 40 °C. This anomaly is consistent with structural changes detected by a distinct endothermic peak at 14 °C using differential scanning calorimetry. Apparently, the optical density anomaly between 10 and 20 °C disappears when the polymorphic transition already has proceeded through previous isothermal equilibration. Thus, melting of equilibrated triglycerides may not affect the RI of milk fat globules at ca. 14 °C as much as melt-mediated polymorphic transitioning. An increased efficiency of UV-C preservation (254 nm) at the translucency optimum was demonstrated by temperature-dependent microbial inactivation experiments.

## Introduction

**Figure 1:**
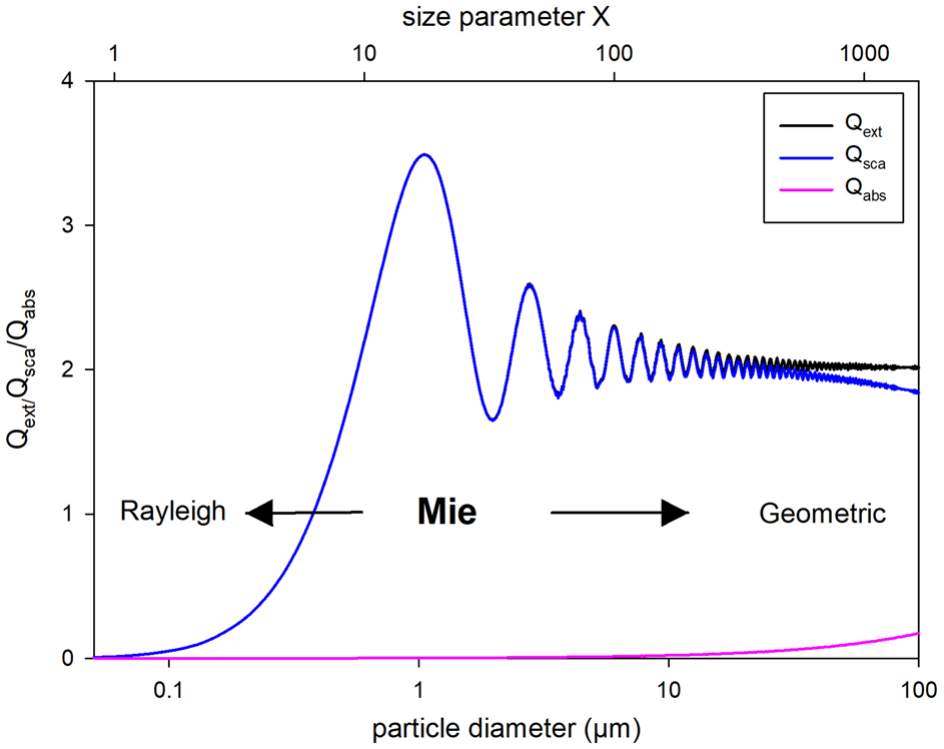
Curves represent calculated extinction (Q_ext_: black), scattering (Q_sca_: blue) and absorption efficiencies (Q_abs_: pink) versus particle diameter (µm) or size parameter *X* (π*d*/λ), respectively, calculated with *Mie Plot v 4.6* (by Philip Laven). Real parts of the refractive index for milk fat droplets were assumed as 1.499 calculated for λ_vacuum_ = 254 nm using equations in Stocker et al.^24^. The imaginary part was kept low at 0.00005, as suggested by Michalski et al. 2001. The refractive index of the serum was assumed as 1.342 (Stocker et al.^24^).

UV-C treatment is a technology of increasing interest to improve or even replace thermal pasteurization, which uses high temperatures to inactivate microorganisms. In contrast to complete heating of the product, UV-C treatment has a more selective effect on individual components such as DNA structures. UV-C treatment irreversibly alters the DNA of microorganisms through the formation of thymine cyclobutene dimers^1–3^. Since UV-C techniques are said to cause a considerably smaller CO_2_ footprint than thermal treatment methods^4–13^, UV-C methods are currently gaining popularity. In addition, UV treatment can also increase the vitamin D concentration of certain foods^14,15^, which is particularly important for the human immune system and bone structure^16–18^. The enrichment of vitamin D_3_ in milk by UV energy is approved by the European Union since 2016^14^. In order to efficiently treat a turbid liquid such as milk with UV energy, the liquid must be mixed during treatment either via turbulent flow or controlled Dean vortices or it needs to be treated as a laminar film flow that is thin enough to be penetrated by UV energy^19^. Especially for the latter, the optical density of the medium is critical. Milk is a complex dispersion of different constituents (e.g., milk fat globules, casein micelles, whey proteins, lactose, and minerals) that influence the optical density of milk. The serum of the milk, which contains whey proteins, lactose, and minerals, is a rather clear medium with a low optical density (Fig. S1). The typical opaque white appearance of milk is caused by scattering of visible light on milk fat droplets (0.8–12 µm in raw milk, < 1 µm in homogenized milk) and on casein micelles (10–800 nm)^20–22^. The ratio of particle size to light wavelength, i.e. the size parameter (*X*, Eq. 1), determines the type of scattering.

1 $$X=\frac{\pi d}{\lambda }$$

where *d* is the diameter of the spherical particles (nm), and *λ* is the wavelength of the incident radiation (nm). Rayleigh scattering occurs at ~ *X* < 1 and geometric scattering at *X* >> 1. For the range in between, Mie scattering applies^23^. Thus, at visible light wavelength (< 700 nm), milk fat globules in homogenized milk are within the Mie region, while the smaller casein micelles may scatter more according to the Rayleigh model. For UV light, both casein micelles and milk fat globules are within the Mie scattering region when dispersed in milk serum (*n*_*serum*_ = 1.342,^24^). In the Mie region, light is scattered more in the forward direction, especially with increasing particle size, in comparison to the isotropic scattering in the Rayleigh region. For a constant incident wavelength, the scattering efficiency (Q_sca_) increases in the Rayleigh region exponentially with particle size (Q_sca_ ~ r^6^). Scattering efficiency is defined as the ratio of the scattering cross section to the geometric cross section. In contrast, in the Mie region, scattering efficiency begins to oscillate with particle size (Fig. 1) due to the complex interference patterns as diffracted and transmitted light interfere. This dependence diminishes with increasing particle size and finally approaches the geometric region (Q_sca_ ≈ 2), which is independent of the particle size (Fig. 1). The exact onset of Mie scattering depends on the particle size and the incident wavelength, but also on the difference between the RI of the particles (milk fat globules, casein micelles) and the surrounding medium (milk serum), since the efficiency curves shift with different RI of the particles. Such a shift can occur, for instance, due to (partial) melting or crystallization of milk fat globules, which would dramatically decrease or increase the refractive index, respectively^25,26^. The melting fractions of milk fat globules are determined by combinations of > 200 different triglycerides^27,28^ resulting in low melting fractions (LMF: < 10 °C), medium melting fractions (MMF: 10–20 °C), and high melting fractions (HMF: > 20 °C). The different triglycerides not only have different melting points, but also exhibit polymorphic behaviour, i.e., at specific temperature (and pressure) they crystallize in phases with the same chemical composition but different crystal structures. Here, the Ostwald Rule of Stages applies for the monotropic polymorphism of milk fat: The least stable orthorhombic γ (or sub-α) form transforming to the metastable hexagonal α form followed by orthorhombic β’ form, and finally reaches the most stable triclinic β form^29,30^. The existence of these polymorphic transitions is clearly proven by X-ray diffraction patterns^29,30^. In this study we expect a reduced RI, i.e., an increased UV light propagation through the milk fat globules during melting/and or melt-mediated polymorphic transitioning, since metastable triglycerides are then temporary disordered, due to dissolution prior to crystallization onto more stable crystals. Therefore, temperature should have a major effect on the scattering type and scattering efficiency of electromagnetic waves on the particles dispersed in milk, not just due to melting but also due to dissociation of metastable crystallized molecules prior to recrystallization (melt-mediated transformation) within the dispersed particles such as milk fat globules. Exact values of RI for milk fat globules at temperatures of 4–40 °C have not yet been identified for UV wavelengths, as well as their influence on light propagation in milk, i.e., optical density, due to different temperatures. Therefore, this study investigates the influence of changes in mild temperatures (4–40 °C) on (1) the optical density of milk and (2) on the associated changes in the efficiency of the UV-C treatment of milk via microbial inactivation experiments at different mild temperatures.

## Results

### Physical properties of milk

**Figure 2:**
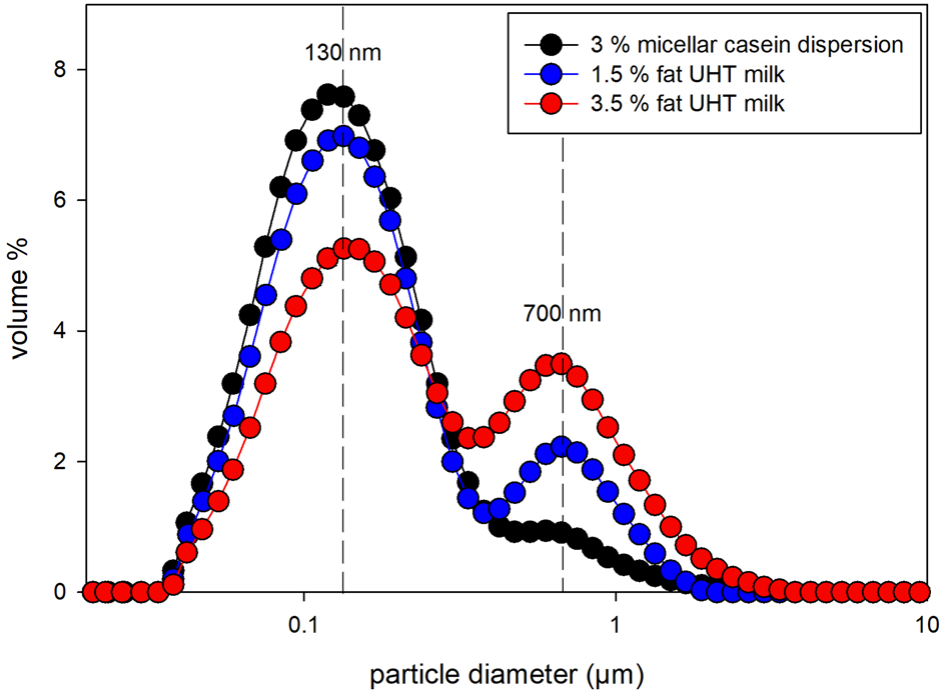
Particle size distribution of the investigated 1.5% and 3.5% fat UHT milk and fat free 3% micellar casein dispersion at 20 °C as volume % vs. particle size measured via laser diffraction with a *Malvern Mastersizer 2000*. Standard deviations (n = 9) are smaller than symbol size. The bimodal distribution of the milk indicates a modal size of 130 nm and 700 nm for casein micelles and milk fat globules, respectively. The almost monomodal peak (120 nm) of the reconstituted casein dispersion agrees with the modal size of casein micelles from milk samples.

**Figure 3:**
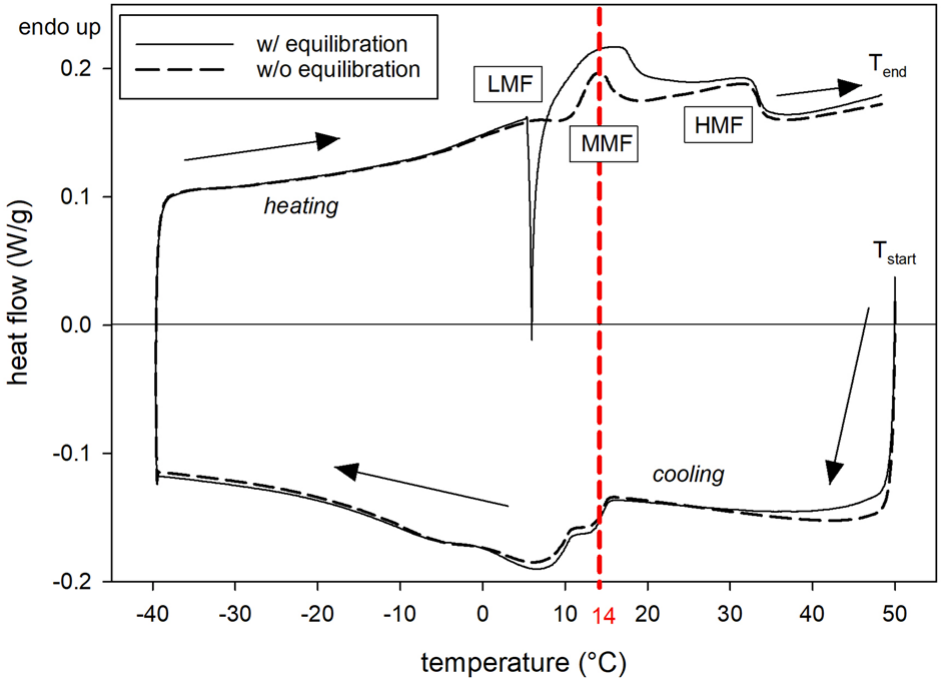
DSC curves recorded at decreasing temperature from 50 to − 40 °C followed by increasing temperature from − 40 to 50 °C of dried 3.5% fat milk with a scanning rate of 5 °C/min. DSC measurements were conducted with and without equilibration at 6 °C for 45 min during recording of the heating. LMF, MMF and HMF indicate the low, medium and high melting fractions caused by different composed triglycerides in milk fat. The distinct endothermic peak at 14 °C may be caused by a melt-mediated polymorphic transitioning from α to β’ triglycerides, while the broad bump of the equilibrated sample rather indicates slow melting of solely β’ triglycerides.

The particle size distribution analyzed via laser diffraction of a homogenized 1.5% and 3.5% UHT (ultra-high temperature processed) milk revealed distinct bimodal distributions (Fig. 2). One peak at approximately 130 nm indicates the mode of casein micelles, while the second peak at approximately 700 nm represents the homogenized milk fat globules. For the 1.5% fat UHT milk the peak at 130 nm is relatively larger when compared to 3.5% fat UHT milk (Fig. 2) indicating an increased casein micelle to fat ratio occurring when reducing the fat content. A fat free micellar casein (3%) dispersion displays an almost monomodal distribution with a peak at 120 nm in good agreement with casein micelles from original milk (Fig. 2). For further characterisation of the UHT milk (3.5% fat) the thermal properties were investigated using differential scanning calorimetry of the dried UHT milk. The analyzed dried milk provides a broad melting range with three main melting fractions (low melting fraction, medium melting fraction and high melting fraction) with the most distinct endothermic peak in the medium melting fraction at 14 °C (Fig. 3), when heating the milk from − 40 to 50 °C after cooling from 50 down to − 40 °C (scanning rate: 5 °C/min). When the milk was equilibrated at 6 °C for 45 min during the heating process, the sharp peak at 14 °C disappears and an almost plateau like bump emerges from 14 to 17 °C.

### Influence of temperature on spectrophotometric results

**Figure 4:**
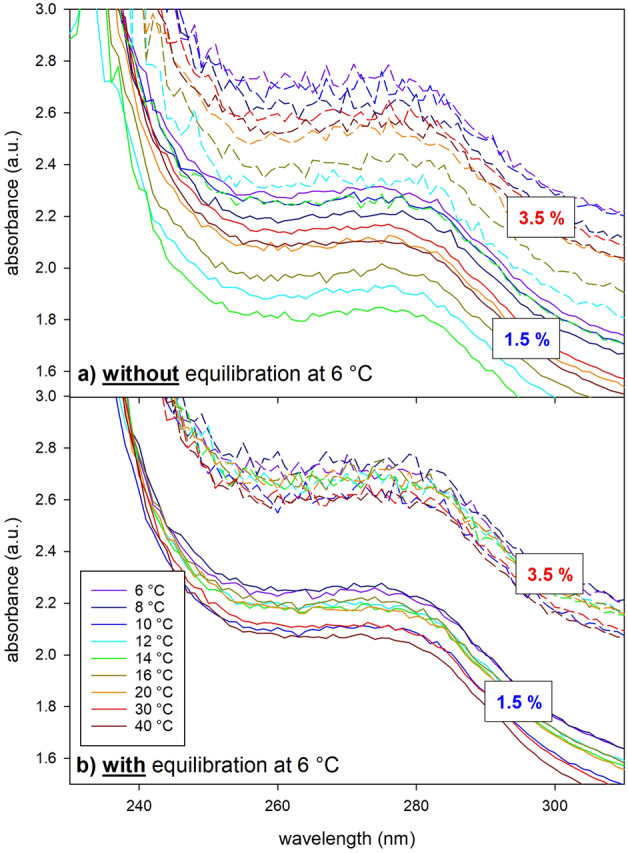
Temperature dependent absorbance spectra of 1.5% (solid lines) and 3.5% fat milk (dashed lines) diluted 1:100 (**a**) without equilibration, i.e., direct analysis after cooling to 6 °C and (**b**) with equilibration at 6 °C, i.e., analysis after 45 min equilibration at 6 °C.

**Figure 5:**
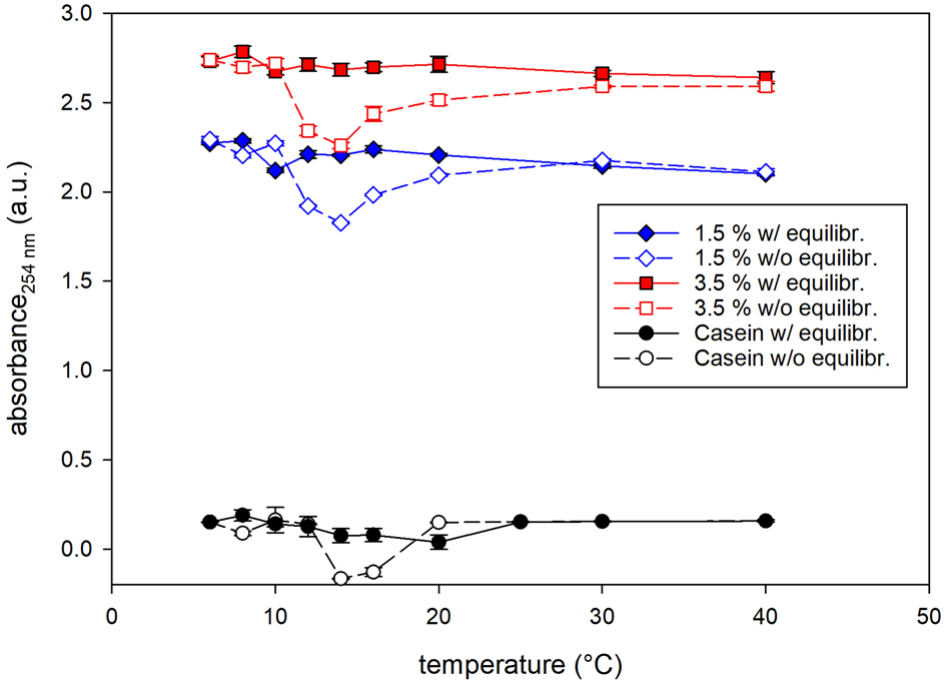
Absorbance at 254 nm vs. experimental temperature. Milks with different fat contents (1.5% and 3.5% fat) and a fat free micellar casein (3%) dispersion equilibrated at 6 °C (ca. 45 min) prior to measurements are shown as filled symbols and solid lines. Samples directly measured without equilibration at 6 °C are shown as open symbols and dashed lines.

Optical spectra were obtained in the UV/Vis range (200–600 nm) as a function of the temperature from 6 to 40 °C for the 1.5% and 3.5% fat UHT milk. Figure 4 shows the results in the UV-range (230–310 nm), which is relevant to UV-treatment of milk. Figure 4a includes the resulting spectra for a sample that was heated and measured immediately after cooling to 6 °C, i.e. without equilibration of the system, while Fig. 4b shows the same experiment with 45 min of equilibration at 6 °C. The non-equilibrated spectra (Fig. 4a) have a wider range with temperature rise, while the equilibrated spectra (Fig. 4b) show less dependence on temperature rise. The optical density data were read at 254 nm and plotted as a function of temperature in Fig. 5. The equilibrated milk shows a steady but marginal decrease in optical density with temperature rise, while the non-equilibrated milk shows an anomaly between 10 and 20 °C with a strong minimum in optical density at 14 °C for both the 1.5% and 3.5% fat UHT milk. Additionally, a fat free micellar casein dispersion (3%) was treated in the same way as the milk samples, i.e., either equilibrated at 6 °C for 45 min or directly heated and measured after cooling to 6 °C (non-equilibrated) (Fig. 5). The casein micelles also show an anomaly in the optical density of the non-equilibrated dispersion in a similar temperature range (12–20 °C) as observed in the milk samples (milk fat globules + casein micelles). However, the occurrence is not as pronounced, since the optical density by the casein micelle dispersion is generally orders of magnitudes lower than by milk.

### Mie scattering of milk fat droplets and casein micelles

**Figure 6:**
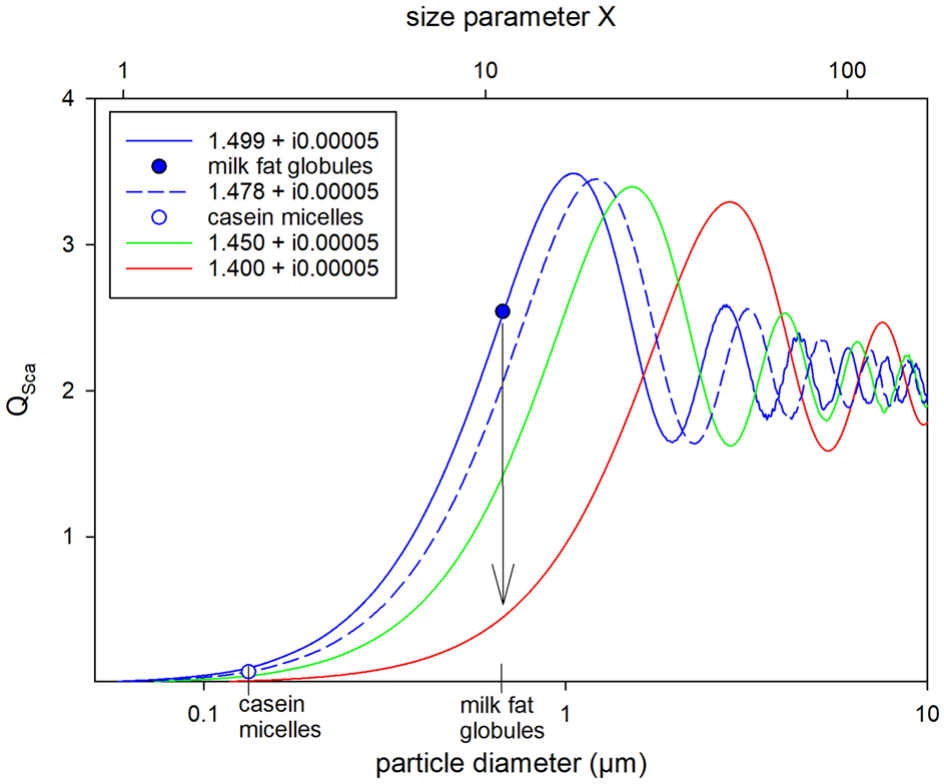
Scattering efficiency (Q_sca_) versus particle diameter (µm) or size parameter *X* (πd/λ), respectively. Curves represent calculated scattering efficiencies calculated with *Mie Plot v 4.6* (by Philip Laven) using the refractive index for milk fat droplets (solid blue: 1.499) and casein micelles (dashed blue: 1.478), calculated for λ = 254 nm using equations in Stocker et al.^24^, as well as two fictive lower values of 1.45 (solid green) and 1.40 (solid red) indicating the influence of melting and/or dissociation of triglycerides in milk fat globules (indicated by arrow). The imaginary part of the refractive index as well as the refractive index of the serum were kept constant at 0.00005^25^ and 1.342^24^, respectively.

Modeling of Mie scattering at 254 nm revealed that even small decreases in the real part of the refractive index would shift the Mie scattering curve toward significantly larger size parameters *X* (Fig. 6). Since both, milk fat globules and casein micelles, are reported to have very small imaginary parts in their refractive indices (i 0.00005)^25^, the scattering efficiency (Q_sca_) is almost equal to the extinction efficiency (Q_ext_) (Fig. 1) and only Q_sca_ is plotted in Fig. 6. Using refractive indices of 1.499 and 1.478 for milk fat globules and casein micelles, respectively, according to Eqs. (2) and (3), and 1.342 as the refractive index of milk serum^24^, the scattering efficiencies by milk fat globules and casein micelles are located in the steep rise of the Mie scattering regime before the oscillating part (Fig. 6). However, it is noteworthy that the scattering efficiency, i.e. the probability for any light interaction, is already very low (Q_sca_ ≈ 0.1) for very small particles like casein micelles (130 nm, i.e., *X* = 2) at a refractive index of 1.478, when compared to Q_sca_ ≈ 2.5 of relatively larger homogenized milk fat globules (700 nm, i.e., *X* = 11) at a refractive index of 1.499. Thus, a hypothetical decrease of the real parts of the refractive indices to, e.g., 1.450 or 1.400 (Fig. 6) due to melting and/or due to dissociation of crystallized molecules within the casein micelles (Q_sca_ ≈ 0.05 and 0.01 for RI = 1.450 and 1.400, respectively) may not affect light propagation as much as aggregate changes within larger milk fat globules (Q_sca_ ≈ 1.4 and 0.5 for RI = 1.450 and 1.400, respectively).

### Temperature effect on microbial UV-C inactivation

**Figure 7:**
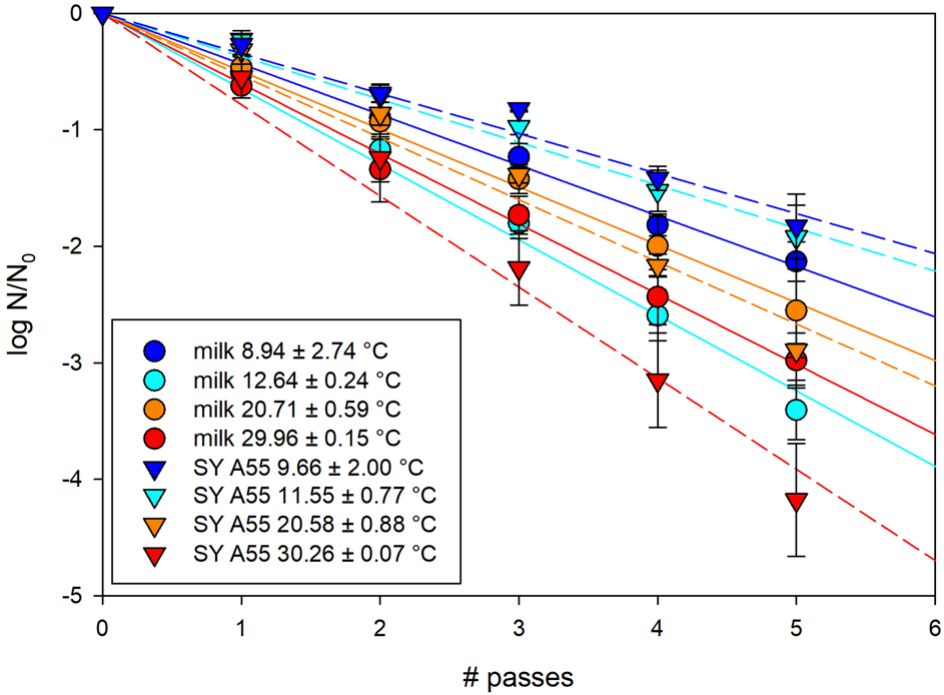
Inactivation of *E. coli* DH5α at different temperatures in 3.5% UHT milk (circles and solid lines) and in a clear model solution (triangles and dashed lines) in a straight tube reactor at laminar flow conditions (30 L/h). The model solution (*sunset yellow FCF* with an absorbance of 55) has at 20 °C a similar inactivation property as 3.5% fat milk, but is a clear solution without particles, i.e. without scattering in the sample.

**Figure 8:**
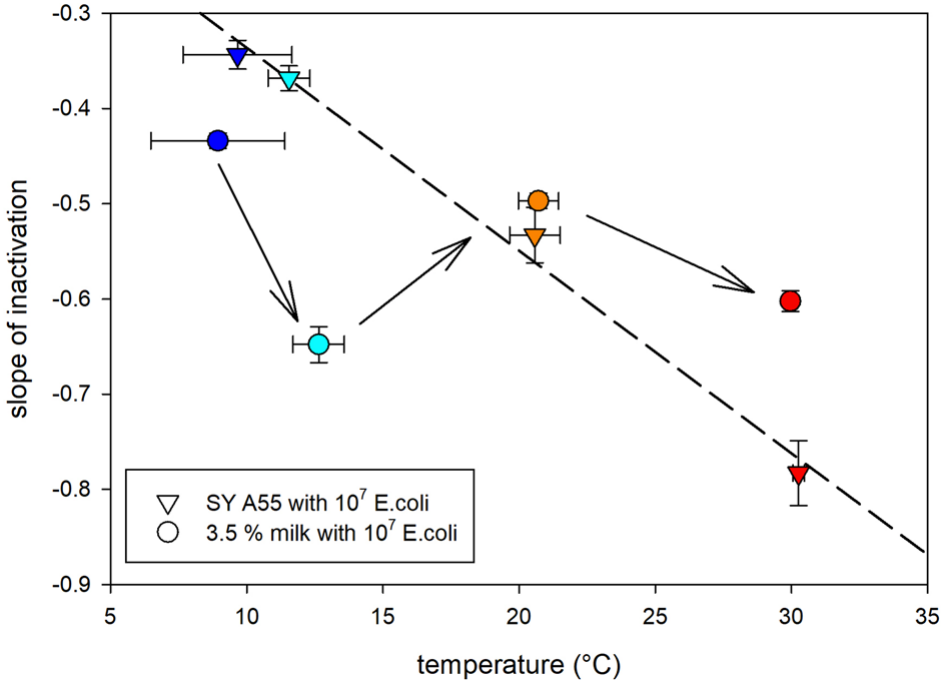
Slope of inactivation of *E. coli* DH5α (from Fig. 7) vs. experimental temperatures in 3.5% fat milk (circles) and in a clear model solution (SY A55) with an absorbance of 55 cm^−1^ at 254 nm (triangles). Temperature of the media were adjusted from room temperature via passing a coil in a crystotate bath before passing through the reactor. Temperature of the medium was kept at target temperature by passing the cryostate bath between each pass through the reactor.

The effect by mild temperature (9–30 °C) on microbial UV-C inactivation was investigated in this study using the 3.5% fat UHT milk and a dyed transparent model solution without any scattering particles. The inactivation curves are plotted for both systems in Fig. 7 and the resulting slopes of each curve were plotted versus temperature in Fig. 8. The inactivation in the transparent model solution increases (negative slope decreases) linearly with increasing mild temperature (Fig. 8). However, when the same experiments were conducted with 3.5% fat UHT milk, containing scattering particles such as milk fat globules and casein micelles, the increase in inactivation efficiency is non-linear (Figs. 7 and 8). Particularly efficient inactivation was observed at 13 °C (Fig. 8). This temperature agrees with the temperature anomalies previously detected by DSC ("Physical properties of milk" section) and optical spectroscopy ("Influence of temperature on spectrophotometric results" section).

## Discussion

Since the particle size distribution as well as the DSC diagram of the here investigated bovine milk agree well with literature data^19–22,27,31^, the here detected results can be discussed in comparison with any other study investigating homogenized bovine milk. The strong endothermic peak detected in this study at ca. 14 °C by DSC (Fig. 3) when heating the milk indicates energy consumption by a strong disordered state of the molecules with a high Gibbs free energy. This disordered state can be either due to melting and/or due to dissolution of metastable crystals such as α triglycerides in milk fat globules prior to recrystallization onto more stable β ‘modifications^29^. For the same cooling/heating rate as used in this study (5 °C/min) Grotenhuis et al.^29^ detected the α and β’ forms between − 18 and 20 °C and 7–35 °C, respectively. Thus, the polymorphic change from α to β’ modification must happen between 7 and 20 °C in accordance with the here detected strong endothermic peak at ca. 14 °C (Fig. 3). Hence, a linkage of this endothermic peak to the polymorphic transitioning from α and β’ modification of the triglycerides in the milk fat globules is probable. In addition, the optical density minimum of 1.5% and 3.5% fat UHT milk detected at 14 °C (Fig. 4a and 5), when immediately heating the milk from 6 to 40 °C, indicates an enhanced UV light propagation through milk. The enhanced translucency of milk disappears for a milk equilibrated at 6 °C for 45 min prior to heating and measurement (Figs. 4b and 5). According to an extrapolation of data by Grotenhuis et al.^29^ a complete solid state transformation from α to β’ modification is expected at isothermal 6 °C after 45 min. Thus, subsequent temperature rise after equilibration would only cause melting of β’ modifications that apparently only marginally decreases the optical density of milk without any distinct minimum (Fig. 5) and also leads to a broad endothermic bump within the DSC diagram instead of a sharp endothermic peak (Fig. 3). These results perfectly fit to observations by Janssen and MacGibbon^32^, who heated milk from 10 to 22 °C soon after cooling it to 10 °C. They detected a significant and sudden decrease in the solid fat content from 25 down to almost 5% prior to an increase up to 15% when milk was heated 5 min after cooling to 10 °C. This minimum disappeared almost completely when the milk was heated 15 min later, i.e., was held at 10 °C for 20 min. Thus, we can conclude, that our observed minimum in optical density at around 14 °C when heating from 6 to 40 °C must be caused by a melt-mediated transformation from metastable α to more stable β’ modification since the temperature of 14 °C is above the melting temperature of α modification (10–12 °C)^32^, while the experiment equilibrated at 6 °C, i.e., below the melting temperature of α modification, led to a solid state transformation from metastable α to more stable β’modifications. Noteworthy, even at 40 °C, where all triglycerides should be melted^33^, the optical density is still significant higher when compared to the optical density minimum detected at 14 °C (Fig. 5). Thus, the melt-mediated transformation from α to β’ modification has a more pronounced effect on the RI of milk fat globules than simple melting of β’ modifications.

Structural changes within casein micelles cannot be the driving force for this translucence anomaly, even if a similar trend (optical density minimum between 12 and 20 °C) is observed in a non-equilibrated fat free (3%) casein dispersion. Its optical density is in general orders of magnitudes lower when compared to 1.5% and 3.5% fat milk (Fig. 5) and its effect on the optical properties of milk may be insignificant. These observations are consistent with the effect expected from modeling of Mie scattering (2.3), where casein micelles should only cause negligible scattering efficiencies, when compared to homogenized milk fat globules at a wavelength of 254 nm (Fig. 6). Thus, the temporary enhanced translucency of milk in the temperature range between 10 and 20 °C must be mainly caused by melt-mediated polymorphic changes from metastable α to more stable β’ modifications of triglycerides within the milk fat globules, lowering its RI and thus its Q_sca_ significantly, which may be superimposed by minor effects occurring in the casein micelles in the same temperature range. The negligible effect of structural changes in casein micelles on the observed temperature dependent anomaly in the optical density of milk may be additionally shown by experiments containing solely dispersed fat globules, which is matter of future research.

The UV-C inactivation experiments clearly demonstrated the direct applicability of a temperature dependent enhanced milk translucency. In contrast to a simple increase of inactivation with increase of mild temperature, as observed in a clear model solution without any scattering particles (Fig. 8) and as also expected from literature^34,35^, the here detected optical density minimum between 10 and 20 °C directly improves the microbial inactivation efficiency in milk in anomalous way. The temperature of the optical density anomaly of about 14 °C may be the ideal temperature to overcome the turbidity of homogenized milk and to treat it with UV-C to increase its shelf life. Since temperature was adjusted between each pass through the UV-C reactor via a coil in a cryostat bath to the target temperature of ca. 12 °C a melt-mediated transition from α to β’ triglycerides may have happened each time when passing the reactor. This repetitive polymorphic transition may lead to the here observed extraordinary effective inactivation between 10 and 20 °C. However, it is noteworthy that a UV-C treatment can likely be further improved by a better temperature control prior to the actual treatment at 14 °C, which was not implemented in this proof of concept of microbial experiments. A rapid cooling (e.g., 5 °C/min) of the milk below 7 °C to achieve only α modification of the triglycerides followed by a rapid rise to 14 °C, where the α form most effectively transitions into the β’ modification via melt mediation, would probably result in the most effective use of the temperature dependent optical density anomaly. Optimizing the temperature profile prior to UV-C treatment at 14 °C is a subject for future research. An improved nutritional value by, e.g., increase of vitamin D concentration via UV-C at this particular temperature is also matter of future research.

## Conclusion

In this study an anomaly in the translucency of milk (1.5% and 3.5% fat) for ultraviolet (UV) light was observed at around 14 °C when heating the medium from 6 to 40 °C. This effect must be caused by a diminishing refractive index due to a strong disordered state within the dispersed milk fat globules. Equilibration of the milk for 45 min at 6 °C prior to heating caused solid state transformation from the metastable α into the more stable β’ modification of the triglycerides, so that subsequent temperature rise would have solely caused melting of the β’ modification. Such a simple melting process only had a marginally effect on the optical density. That is, the temperature dependent anomaly in optical density observed in the non-equilibrated milk must be caused by melt-mediated transformation from the metastable α to the more stable β’ form of the triglycerides. Such a temperature dependent anomaly in milk can be directly utilized for preservation by UV-C techniques as proven by microbial inactivation experiments in milk at different mild temperatures presented in this study.

## Methods

### Samples

The milk samples measured in this study by different analytical methods were drawn all from one batch of 3.5% and 1.5% fat UHT milk, respectively, supplied by *frischli Milchwerke GmbH*. A fat free micellar casein dispersion was mixed from 3% microfiltrated micellar casein powder with > 88% pure, native milk proteins (*Würzteufel GmbH, Gewürzmanufaktur Schwarzwald*). The powder was mixed with a saline solution (0.2% NaCl) since reconstitutability of casein micelles is enhanced by the presence of NaCl^36^ using an *Ultra Turrax T25* equipped with *GS 25 N-18* dispersing tool for five minutes (13.500 rpm) with subsequent 5 min centrifugation using a *Sorvall RC26 plus* with rotor *SLA 3000* (6.500 rpm) to remove remaining large particles. The resulting particle size distribution after this procedure was most similar (~ 120 nm) to original casein micelles (~ 130 nm) (Fig. 2). A model solution for microbial inactivation experiments was dyed with the food colorant *Sunset Yellow FCF* in order to get a transparent model solution excluding any scattering particles. The concentration of *Sunset Yellow FCF* was adjusted to gain an absorbance of 55 cm^−1^ of the solution. This absorbance was chosen as at 20 °C *E. coli* DH5α is inactivated in this model solution similarly effective as in 3.5% fat UHT milk (Fig. 7).

### Size analysis via laser diffraction

The milk samples as well as the fat free micellar casein dispersion used for the experiments were investigated on their particle size distribution using laser diffraction in a *Hydro2000SM* measurement cell (at 1500 rpm) of the *Malvern Mastersizer 2000* (software version 6.00). Refractive indices were taken from Michalski et al.^25^ with 1.33 for the dispersant (water) and 1.458 (633 nm) or 1.46 (466 nm), respectively, for the particles as well as the particle absorption indices with 5 × 10^–6^ (633 nm) and 1.7 × 10^–5^ (466 nm). For the measurements the milk was diluted with room temperature (~ 20 °C) water as the refractive indices by Michalski et al.^25^ are valid for this temperature. The particle size model was set on “general purpose” and the particle shape was set on “spherical”, as both casein micelles and milk fat globules are assumed to be relatively perfect spheres. Three samples were taken from each milk charge (1.5% and 3.5% fat) and were measured as threefold, i.e., each datapoint relies on nine measurements.

### DSC measurement

Differential scanning calorimetry (DSC) curves were recorded using a differential scanning calorimeter *Q2000 v24.11* (*TA-Instruments-Waters LLC*) equipped with a refrigerated cooling system (*RCS90*). Data analysis was performed with *Universal Analysis 2000 ver. 4.5A* software. Nitrogen was used as purge gas with a flow rate of 50 mL/min. Temperature calibration of the instrument was carried out for all applied cooling and heating rates and the Tzero pan using an indium reference material (melting point:156.61 °C, enthalpy of fusion: 28.706 J/g). The whole milk samples were dropped and dried stepwise onto a Tzero aluminum pan reaching sample weights of 9.13 ± 0.97 mg. The pans were only crimped and not hermetically sealed allowing possibly remaining water to escape the pan during initial heating to 50 °C. As reference an empty sealed Tzero pan was used. Prior to the actual analysis the samples were heated to 50 °C to eliminate all crystal nuclei. Then the temperature was decreased down to − 40 °C followed by heating up to 50 °C, both with a scanning rate of 5 °C/min according to literature^29,31^. The heating was recorded with and without equilibration at 6 °C for 45 min. The experiments were performed in triplicates, but only one experiment of each procedure is here shown for a better clarity.

### Spectrophotometric measurements

Using a spectrophotometer (*UNICAM UV2-100 UV/VIS* spectrometer) the absorbance in the UV–Vis range (200–600 nm) was measured of the here investigated milk (1.5% and 3.5% fat), whey (from 3.5% fat milk) and model solutions (dyed with *Sunset Yellow FCF*) diluted 1:100 with DI water. Temperature dependent optical spectra were obtained with an *Analytik Jena Spektralphotometer Specord 210 Plus* (photometric measuring range from − 3 to 3) equipped with a thermostat bath tempering the cuvettes. The samples (1.5% and 3.5% fat UHT milk as well as a fat free 3% micellar casein powder dispersed in saline solution) were mixed 1:100 with room temperature DI water and cooled in a fridge down to 6 °C. Once this temperature was reached the samples were placed into the precooled spectrometer. The samples were either equilibrated for 45 min at 6 °C or directly stepwise heated with approximately 0.2 °C/min. Spectra were recorded at 6, 8, 10, 12, 14, 16, 20, 30 and 40 °C. All samples were measured in triplicates in disposable semi-micro UV cuvettes with an optical path length of 10 mm.

### Modelling of Mie scattering

Scattering, extinction and absorption efficiencies were calculated using the open access software *Mie Plot v 4.6* (by Philip Laven). Wavelength of incident irradiation and the refraction index (RI) of the surrounding medium (milk serum) were kept constant at 254 nm (in vacuum) and 1.342^24^, respectively. The according real parts of RI were calculated using the Cauchy’s equations Eqs. (1) and (2) after Stocker et al.^24^ for milk fat globules and casein micelles, respectively.

2 $${RI}_{fat}\left(\lambda \right)=1.4505+3.157\cdot \frac{{10}^{3}{\mathrm{nm}}^{2}}{{\lambda }^{2}}+1.0654\cdot \frac{{10}^{4}{\mathrm{nm}}^{4}}{{\lambda }^{4}}$$

3 $${RI}_{casein}\left(\lambda \right)=1.38+6.83\cdot \frac{{10}^{3}{\mathrm{nm}}^{2}}{{\lambda }^{2}}-0.33\cdot \frac{{10}^{8}{\mathrm{nm}}^{4}}{{\lambda }^{4}}$$

The empirical values in Eq. (3) were taken from the fitting of the scattering coefficient values μ_s_ collected with collimated transmission measurements from Stocker et al.^24^ instead from the fitting of the reduced scattering coefficients μ’_s_ that were measured with an integrating sphere^24^. At visual light (> 400 nm), as used in previous publications, both values may be applied successfully to evaluate the RI for casein micelles, as such relatively long wavelength cause rather isotropic scattering with anisotropy factors (g) < 1.

4 $${\mu {\prime}}_{s}={\mu }_{s}\cdot (1-g)$$

Equation (4) shows the connection between the reduced scattering coefficient μ’_s_ and the scattering coefficient μ_s_, where *g* is the anisotropy factor. However, at UV wavelength, such as 254 nm, the anisotropy and thus g of casein micelles increase to ~ 1. For particles causing such a high anisotropy RI cannot be approximated accurately from μ’_s_, as the reduced scattering coefficient will significantly underestimate the amount of scattering. Consequently, μ_s_ and not μ’_s_ should be taken to calculate RI of casein micelles at deep UV-wavelength. Further, the scattering coefficient μ has a better reproducibility for casein than μ’_s_ according to Postelmans et al.^37^. Using Eqs. (2) and (3) to estimate RI at λ = 254 nm results in 1.499 and 1.478 for the real parts of milk fat globules and casein micelles, respectively. Noteworthy, the solution of Eq. (2) for λ = 633 and 466 nm result in *RI*_*fat*_ = 1.458 and 1.465, respectively, in agreement with the values suggested by Michalski et al.^25^. For simplification the imaginary parts are kept constant at a low value of i0.00005, as very low absorption is assumed for milk fat globules and casein micelles having a negligible effect on light propagation in milk^24,25^.

### UV-C inactivation of E.coli in milk and model solution

All UV-C experiments were conducted in the commercially available UV box (BS04 UV box; UV Messtechnik Opsytec Dr. Gröbel GmbH, Ettlingen, Germany) containing twenty 18 W low-pressure mercury lamps with a specified UV-C output of 4.5 W for each lamp. Here, a tube system consisting out of 24 straight tubes with an inner diameter of 6 mm and an outer diameter of 6.6 mm was installed ca. 300 mm below the UV-C lamps. The tubes consist of UV-C transparent fluorethylenpropylen (FEP) and are connected with non-UV-transparent U-turns. The total length is 19.3 m of which 16.1 m can be penetrated by the UV light. The distance between tubes and lamps allows an investigation of the temperature effect on UV-C efficiency without influencing the efficiency of the lamps. The sample solutions were tempered during the experiments at approximately 9, 12, 20 and 30 °C using a coil in a cryostat bath (F32; Julabo GmbH, Seelbach, Germany). Differences in inactivation efficiency caused by changes in optical density (due to temperature) of the media will be more significant, when medium passes the reactor with minimal mixing. Thus, the volume flow of the media was adjusted to 30 L/h using a peristaltic pump (Heidolph, Pumpdrive S206) in order to keep Reynolds number *Re* < 2300, i.e., at laminar flow conditions. One hour before starting the experiment the lamps were switched on to reach a constant UV-C irradiation. The system was cleaned and sanitized with 70% ethanol prior to each experiment. The samples were pumped five times completely through the reactor, i.e. five passes. To prevent mixing with sample material that remained in the device from the prior pass > 150 mL of each pass was discarded, before sampling.

In this study *Escherichia coli* DH5α (obtained from the strain collection of the Department of Food Technology and Bioprocess Engineering at MRI) was used to investigate the inactivation efficiency by UV-C irradiation at different temperatures. *Escherichia coli* DH5α (*E. coli*) is a common and safe gram-negative test bacterium and is representative of the enterohemorrhagic *E.coli* spp.^38^ in the experiments. Gram-negative bacteria are frequently present in dairy products associated with reduced hygiene and can reach a very high cell count. *E. coli* was grown from cryovials and sub-cultured twice before it was used in the experiments. *E. coli* was incubated each time at 37 °C for 18 h in Std I nutrition medium using a shaking incubator (Standard Analog Shaker stage at 150 rpm). For the experiments 3.5% fat UHT milk or dyed (*Sunset Yellow FCF*) model solution without scattering particles was inoculated with the *E. coli* suspension at a concentration of approximately 10^7^ cfu/mL. To allow acclimatization of the microorganisms, the inoculated sample was incubated at room temperature for 20 min before the experiments. To determine the count of the microorganisms before UV-C treatment and after each pass of the experiments, samples were taken accordingly and diluted using the ten-fold dilution method and incubated on Std I agar medium for 18 h. Each dilution of each pass was plated two to four times. Pictures were taken with a camera and the software ImageJ was used to count colonies. When possible, an automatic colony counting method^39^ was applied. The results of each dilution step were averaged. For statistical validation all experiments were conducted in triplicates.

### Supplementary Information

Supplementary Figure S1.

## Data Availability

All data generated or analyzed during this study are included in this published article (and its Supplementary Information files). Raw data generated during and/or analyzed during the current study are available from the corresponding author on reasonable request.
