# Supplementary material for: Observation of a temperature dependent anomaly in the UV translucency of milk useful for UV-C preservation techniques
Source: Sci Rep. 2023 Dec 11;13:21937. doi: 10.1038/s41598-023-49124-y (PMC10713634; doi:10.1038/s41598-023-49124-y)
Supplement: Supplementary file 1 — Supplementary Figure S1. [file 41598_2023_49124_MOESM1_ESM.pdf]

Supplementary of

"How temperature affects the translucency of milk and its ability for UV-C treatment "

Jaayke Fiege[1], Benedikt Woll[1], Stefan Hebig[1], Alexandra, Dabrowski[2], Volker Gräf[1], Elke Walz[1], Steafn Nöbel[2], Katrin Schrader[2], Mario Stahl[1]

[1] *Department of Food Technology and Bioprocess Engineering, Max Rubner-Institut, Federal Research Institute of Nutrition and Food, D-76131 Karlsruhe, Germany*

[2]*Department of Safety and Quality of Milk and Fish Products, Max Rubner-Institut, Federal Research Institute of Nutrition and Food, D-24103 Kiel, Germany*

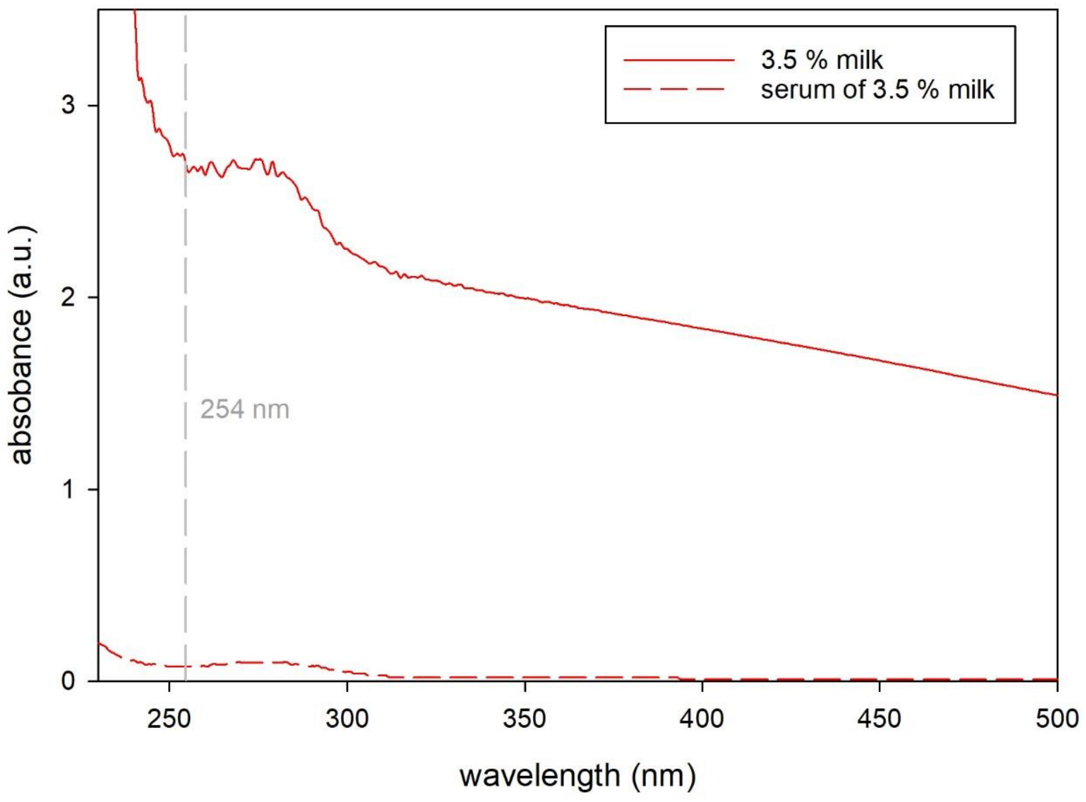

Fig. S1: Absorbance versus wavelength of 3.5 % whole milk and serum made from acidification of this whole milk. Both liquids were diluted 1:100 to record the absorbance spectra. The UV-C wavelength of 254 nm is highlighted.
